# Effects of Meal Frequency on Metabolic Profiles and Substrate Partitioning in Lean Healthy Males

**DOI:** 10.1371/journal.pone.0038632

**Published:** 2012-06-13

**Authors:** Marjet J. M. Munsters, Wim H. M. Saris

**Affiliations:** Department of Human Biology, Nutrition and Toxicology Research Institute Maastricht (NUTRIM), Maastricht University Medical Center (MUMC+), Maastricht, The Netherlands; University of Tübingen, Germany

## Abstract

**Introduction:**

The daily number of meals has an effect on postprandial glucose and insulin responses, which may affect substrate partitioning and thus weight control. This study investigated the effects of meal frequency on 24 h profiles of metabolic markers and substrate partitioning.

**Methods:**

Twelve (BMI:21.6±0.6 kg/m^2^) healthy male subjects stayed after 3 days of food intake and physical activity standardization 2×36 hours in a respiration chamber to measure substrate partitioning. All subjects randomly received two isoenergetic diets with a Low meal Frequency (3×; LFr) or a High meal Frequency (14×; HFr) consisting of 15 En% protein, 30 En% fat, and 55 En% carbohydrates. Blood was sampled at fixed time points during the day to measure metabolic markers and satiety hormones.

**Results:**

Glucose and insulin profiles showed greater fluctuations, but a lower AUC of glucose in the LFr diet compared with the HFr diet. No differences between the frequency diets were observed on fat and carbohydrate oxidation. Though, protein oxidation and RMR (in this case SMR + DIT) were significantly increased in the LFr diet compared with the HFr diet. The LFr diet increased satiety and reduced hunger ratings compared with the HFr diet during the day.

**Conclusion:**

The higher rise and subsequently fall of insulin in the LFr diet did not lead to a higher fat oxidation as hypothesized. The LFr diet decreased glucose levels throughout the day (AUC) indicating glycemic improvements. RMR and appetite control increased in the LFr diet, which can be relevant for body weight control on the long term.

**Trial Registration:**

ClinicalTrails.gov NCT01034293

## Introduction

The escalating obesity trend in man is due to an imbalance between energy intake and energy expenditure. Energy intake is influenced by the effect of food’s energy density, total energy content and meal frequency and the extent to which these alter satiety. Of these factors, meal frequency has received least attention [1].

Epidemiological evidence indicates increasing trends in recent years of dietary snacking and increased meal frequency [2], [3]. The current literature is mixed with regard to the efficacy of increased meal frequency (or snacking) regimens in causing metabolic alterations, particularly in relation to weight management [1]. Increasing eating frequency has been postulated to increase metabolism, reduce hunger and food cravings (better appetite control), improve glucose and insulin control, and reduce body weight and body fat storage [4]. However, there are suggestions from experimental studies to date as well as from cross-sectional epidemiological studies, in which energy intake underreporting is taken into account, that greater eating frequency (snacking) may promote positive energy balance in free-living adults [5], [6]. On the other hand, well-controlled intervention studies do not support an association between eating frequency and body weight [6], [7].

Eating three meals a day is suggested to result in a higher postprandial insulin peak due to the higher carbohydrate (CHO) intake and thereby increasing cellular glucose uptake and oxidation. As a consequence, dietary fat is primarily stored in the adipose tissue (insulin stimulated activation of lipo-protein lipase) during the postprandial phase. In between meals, the fasting state, when insulin levels are decreased and lipolysis is activated this substrate flux is reversed [8], [9].

Given the inconclusive evidence in the literature regarding meal frequency and its metabolic implications, very well-controlled trials are necessary to resolve speculation that the current increase in snacking habits contribute by its metabolic changes during the day to the escalating obesity epidemic. For that reason, the aim of the present study was to investigate the mechanistic effects of meal frequency on 24 hr insulin, glucose profiles, appetite profiles and substrate partitioning under well-controlled energy balance conditions. We hypothesized that in an energy balanced situation eating 3 meals a day gives better opportunities to turn the metabolic flux into a prolonged fasting state with a higher fat oxidation compared to eating 14 meals a day where subjects remain in a continuous postprandial status.

## Materials and Methods

The protocol for this trial is available as supporting information; see Protocol S1.

**Table 1:** Subject characteristics at baseline.

Subject characteristics	Group (n = 12)
Age (yrs)	23±1.2(18–31)
BMI (kg/m^2^)	21.6±0.6 (19.1–24.6)
Fat free mass (kg)	62.1±1.3 (52.1–67.8)
Body fat (%)	14.1±1.4 (5.1–21.6)
Systolic blood pressure (mm/Hg)	114±2.9 (98–133)
Diastolic blood pressure (mm/Hg)	69±2.3 (59–83)
Fasting glucose level (mmol/L)	5.2±0.1 (4.8–5.7)
2 h glucose level after OGTT (mmol/L)	4.1±0.3 (3.2–7.1)
Fasting insulin level (mU/L)	13.4±2.9 (5.9–44.6)
OGIS_120_ (ml/min^/^.m^2^)	455.1±8.5 (396–495)
HOMA_IR_	2.4±0.7 (1.3–11.2)

Values are expressed as mean±SEM.

### Study Population

The study was conducted between 21^th^ October 2009 and 19^th^ March 2010 on 12 adults. Subjects were recruited by advertisements at local educational institutions. All subjects were healthy as assessed by a medical history questionnaire, blood pressure measurement and an Oral Glucose Tolerance Test (OGTT). Subjects had to be weight stable over the past 3 months. Exclusion criteria were: BMI>25 kg/m^2^; metabolic abnormalities and excess alcohol intake (>28 drinks weekly). Given that energy expenditure declines with increasing age, a maximal age of 40 years was set to form a homogeneous adult group. Only males were included to avoid menstrual cycle effects on energy expenditure. In addition, only males of European descent were included for homogeneity reasons. This study was conducted according to the guidelines laid down in the Declaration of Helsinki [10]. The Medical Ethical Committee of the University Hospital Maastricht approved all procedures involving human subjects. Written informed consent was obtained from all subjects.

**Table 2:** 24h total energy intake and expenditure, components of energy expenditure and substrate partitioning, according to Low or High Frequency (LFr and HFr diet resp.) diet.

	LFr diet	HFr diet	p-value^1^
EI (MJ/d)	12.0±0.3	12.0±0.3	-
TEE (MJ/d)	12.3±0.3	12.1±0.3	0.122
EB (MJ/d)	-0.4±0.2	-0.1±0.1	0.116
RMR (MJ/d)	8.5±0.3	8.0±0.2*	0.006
SMR (MJ/d)	7.2±0.2	7.0±0.2	0.154
DIT (MJ/d)	1.3±0.1	1.0±0.1	0.094
AEE (MJ/d)	3.8±0.2	4.1±0.2	0.238
PAI	1.72±0.03	1.73±0.03	0.570
RQ	0.91±0.01	0.91±0.01	0.658
Protein oxidation (g/d)	106.9±7.1	90.6±4.3*	0.021
Carbohydrate oxidation (g/d)	455.5. ±16.3	456.8±17.4	0.946
Fat oxidation (g/d)	61.9±5.2	64.6±4.6	0.647

EI: energy intake. TEE: total energy expenditure. RMR: resting metabolic rate. DIT: diet induced thermogenesis. AEE: activity-induced energy expenditure. PAI: physical activity index. RQ: respiratory quotient.

Values are expressed as mean±SEM. *P<0.05 compared with the LFr diet.

1 P-values were derived by paired t-test analysis and denote the overall significance of differences among the two diets.

### Screening

All subjects performed an OGTT before inclusion. Subjects came to university in the morning after an overnight fast. A catheter was placed into an antecubital vein and a fasting blood sample was taken. Next a bolus of 75 g glucose (dissolved in 250 ml water) was ingested (t = 0 min). Blood was sampled every 30 minutes until t = 120 min. Plasma glucose levels were measured to determine glucose tolerance. In addition, plasma glucose and insulin levels were used to assess insulin sensitivity using the oral glucose insulin sensitivity (OGIS)-index for a 2 h OGTT as described by Mari et al [11]. Insulin sensitivity in the basal state was determined by the homeostasis model assessment insulin resistant **(**HOMA_IR_) [12].

**Figure 1:**
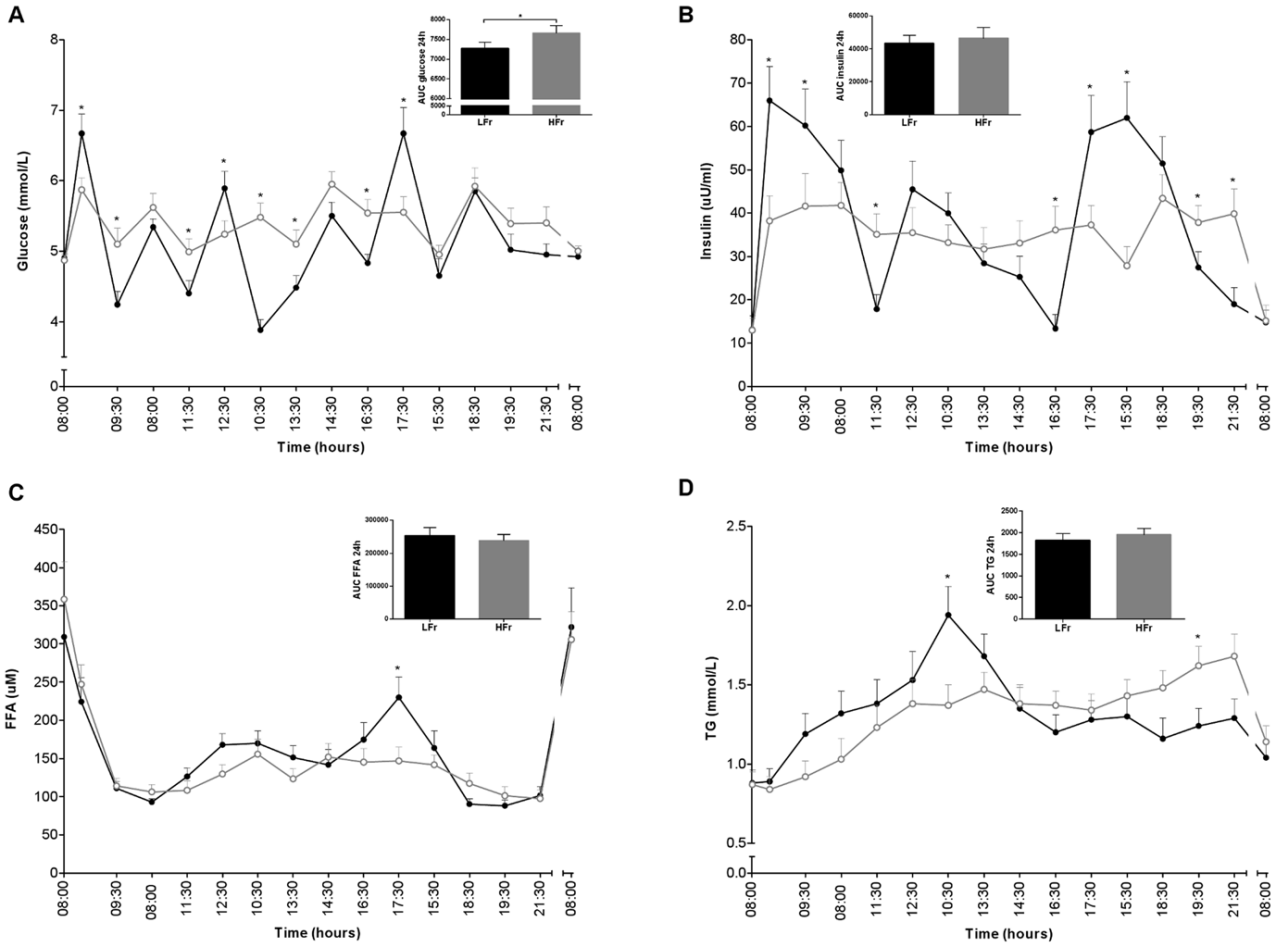
Glucose (A), insulin (B), FFA (C) and TG (D) levels for 24 h and the AUCs of the LFr (dense black circle) and HFr (open gray circle) diet. *P<0.05 LFr vs. HFr diet. P-values were derived by analysis of mixed models for the 24h profiles and by a paired t-test for the AUCs. ^a^Values are expressed as mean±SEM.

**Figure 2:**
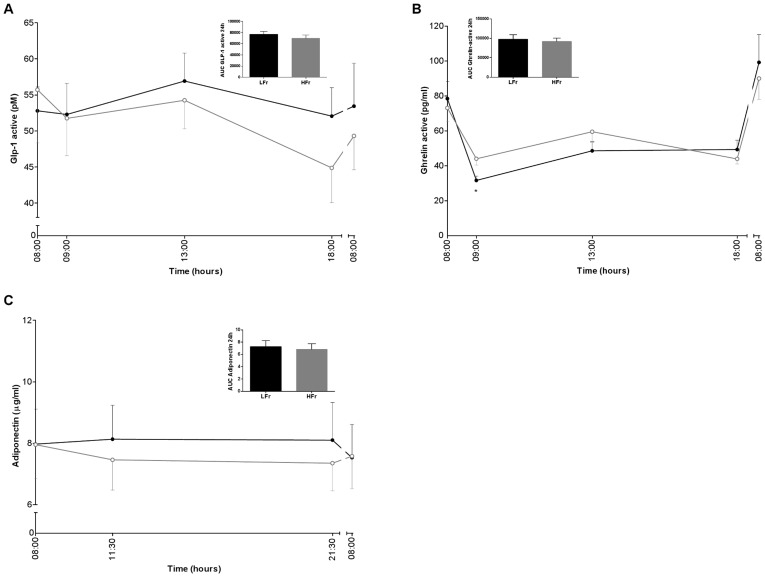
GLP-1 active (A), ghrelin-active (B) and adiponectin (C) levels for 24 h and the AUCs of the LFr (dense black circle ) and HFr (open gray circle ) diet. *P<0.05 LFr vs. HFr diet. P-values were derived by analysis of mixed models for the 24 h profiles and by a paired t-test for the AUCs. ^a^Values are expressed as mean±SEM.

### Study Design

This study had a randomized, 2-way crossover design with a wash-out period of at least one week to avoid interaction between the two interventions. Each intervention lasted 36 hours in the respiration chamber [13]. All subjects randomly received the same diet with a Low meal Frequency (3×; LFr) or a High meal Frequency (14×; HFr) with a constant macronutrient composition of 15 energy% (En%) dairy protein, 30 En% fat, and 55 En% carbohydrates in each meal. The protein consisted of 70% dairy protein and 30% vegetable protein. The diet was composed of LU cracottes natural (210 g), milk (semi-skimmed;1540 ml), yoghurt drink (Vifit natural;200 ml), melon (1500 g), tomato (640 g) and olive oil (47 ml) for a 2400 kcal diet. The LFr diet consisted of breakfast at 08.00 h, lunch at 12.00 h, and dinner at 17.00 h. In the HFr diet meals were consumed every hour from 08.00 h until 21.00 h. The choice of 14 meals was made to ensure that subjects were in a continuous postprandial status and in the range of the published high meal frequencies studies [1]; six to seventeen.

**Table 3:** Analyses of the CGMS data compared between the two intervention diets (n = 12).

	LFr diet	HFr diet	p-value^1^
Mean 24 h (mmol/L)	4.6±0.1	4.7±0.2	0.614
Min 24 h (mmol/L)	3.7±0.2	3.6±0.2	0.711
Max 24 h (mmol/L)	5.6±0.2	5.6±0.2	0.906
Mean 8–23 h(mmol/L)	4.7±0.1	4.8±0.2	0.648
Min 8–23 h (mmol/L)	3.8±0.2	3.7±0.2	0.782
Max 8–23 h (mmol/L)	5.6±0.2	5.6±0.2	0.806
Mean 23–8 h(mmol/L)	4.5±0.2	4.6±0.1	0.587
Min 23–8 h (mmol/L)	4.0±0.2	4.0±0.2	0.810
Max 23–8 h (mmol/L)	4.9±0.2	5.2±0.2	0.249
AUC 24 h	6657.3±198.7	6759.1±218.5	0.601
AUC 8–23 h	4230.7±118.3	4288.8±149.1	0.646
AUC 23–8 h	2448.1±93.3	2492.3±74.9	0.584
net iAUC 24 h	525.3±242.3	855.1±223.3	0.191
net iAUC 8–23 h	398.3±130.1	598.8±131.6	0.163
net iAUC 23–8 h	108.1±64.3	80.3±79.5	0.777
CONGA1	0.43±0.05	0.34±0.02	0.158
CONGA2	0.45±0.06	0.39±0.02	0.634
CONGA4	0.53±0.07	0.46±0.03	0.565
CV	0.09±0.01	0.08±0.01	0.999

Min: minimal glucose level. Max: maximal glucose level. AUC: area under the curve. net iAUC: net incremental area under the curve. CONGA1,2,4: continuous overall net glycemic action describing intra-day glycemic variability between respectively 1,2 and 4 h time periods over 24 h. CV: coefficient of variability.

^a^Values are expressed as mean±SEM. *P<0.05 compared with the LFr diet. ^1^P-values were derived by paired t-test analysis and denote the overall significance of differences among the two diets.

Subjects were allowed to consume water and tea after 18.00 h *ad libitum*, because after that time point no VAS questionnaires had to be completed. Subjects were fed isoenergetic based on the individual energy requirements. For measurement and calculation see description respiration chambers. Subjects standardized their food intake and activity for 3 days before each test to have the same baseline condition. Food-intake and activity diaries had to be filled out before the first test and subjects were instructed to follow the same regime preceding the second test.

**Figure 3:**
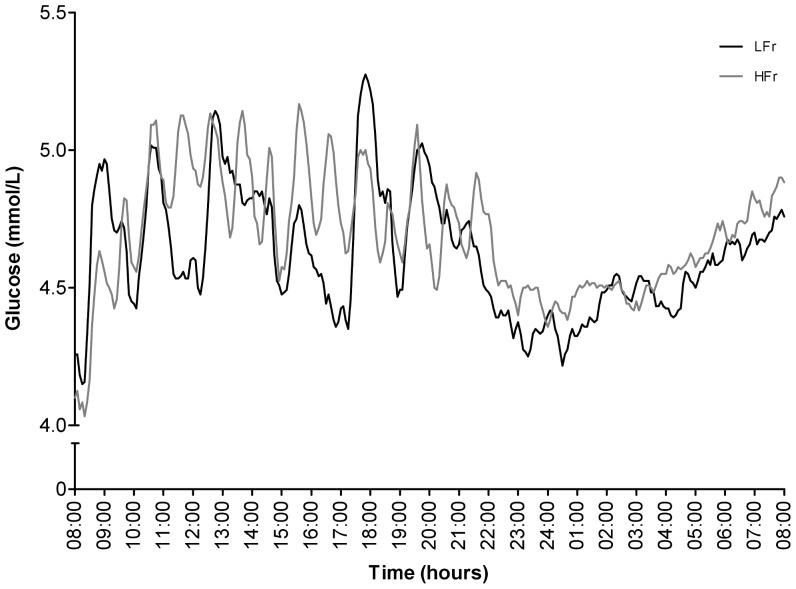
CGMS glucose levels for 24 h in the LFr and HFr diet. ^a^Values are expressed as mean.

Subjects entered the respiration chamber at 20.00 h and finished the intervention 36 h later at 8.00 h. Physical activity was prescribed by means of a standardized physical activity protocol, three times of stepping (15 minutes). It was carefully controlled that subjects were fed in energy balance, which was based on individually measured and calculated requirements. During the first night in the respiration chamber the sleeping metabolic (SMR) rate was assessed. The SMR is defined as the lowest energy expenditure (measured in 30 minutes intervals) over three consecutive sleeping hours. Based on the SMR (multiplied with a physical activity index (PAI) of 1.55), the total daily energy expenditure (EE) was estimated [14]. This level of EE was used subsequently as energy intake level for both 24 h intervention days in the respiration chamber.

**Figure 4:**
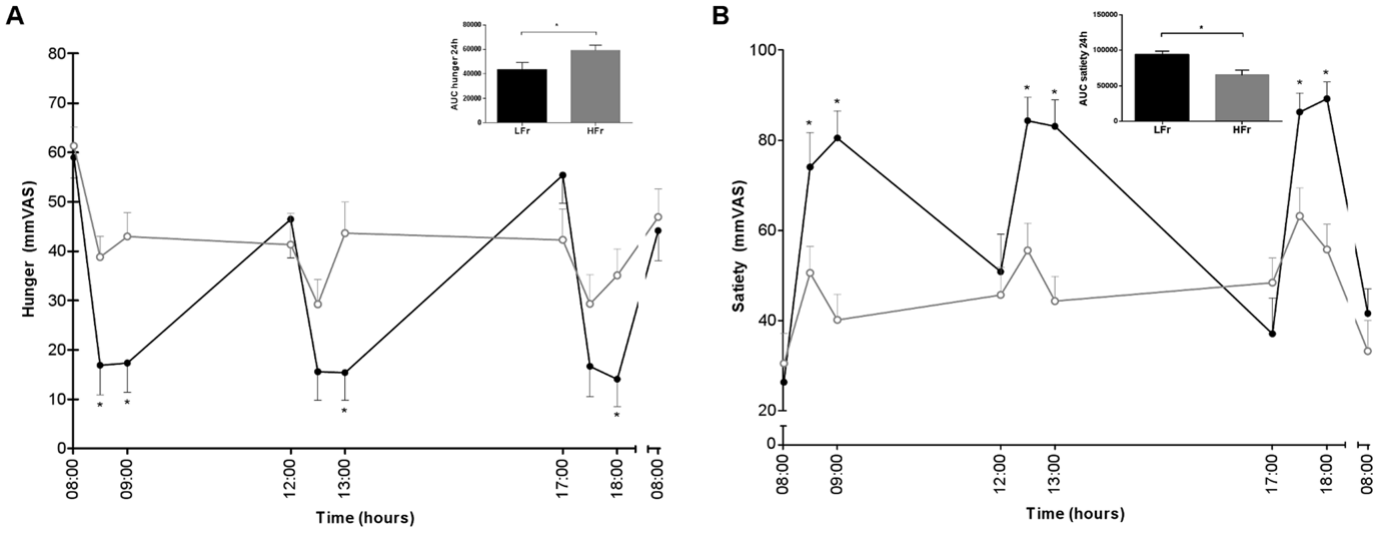
Hunger (A), and satiety (B) levels for 24 h and the AUCs of the LFr (dense black circle ) and HFr (open gray circle ) diet. *P<0.05 LFr vs. HFr diet. P-values were derived by analysis of mixed models for the 24 h profiles and by a paired t-test for the AUCs. ^a^Values are expressed as mean±SEM.

### CGMS

Each respiration chamber visit started after placement of a MiniMed sensor and MiniMed Continuous Glucose Monitoring System Gold (CGMS™) to measure subcutaneous interstitial fluid glucose levels over 36 h (Medtronic Minimed, Northridge, USA). The glucose monitor sampled the signals once every 10 seconds and recorded an average signal every five minutes, providing as many as 288 Sensor readings in a 24 h period. The monitor was calibrated with four separate capillary finger prick glucose readings using a glucose meter (Glucocard Memory PC; A. Menarini Diagnostics, Florence, Italy). Although the sensors recorded data for 36 h, only the last 24 h data (monitored between 08.00 hours on day 2 and 08.00 hours on day 3) were used for analysis. Continuous overall net glycemic action (CONGA), a novel method described by McDonnell et al [15], was used to assess intra-day glycemic variability. CONGA*n* was defined as the standard deviation of the differences in glucose concentration between current observation and the observation *n* hours previous. CONGA1, CONGA2, and CONGA4, were calculated indicating intra-day glycemic variability based on 1 h, 2 h and 4 h time periods. Higher CONGA values indicate greater glycemic variation, values above 1.5 indicate glycemic lability [15]. The coefficient of variability (CV) is defined as the SD divided by the mean of the glucose values [16].

### Blood Sampling

In the morning, while staying in the chamber, a catheter was placed into an antecubital vein using a airtight sleeve for the withdrawal of blood. Blood was sampled just before ingestion of the first meal (baseline), 30 minutes postprandially, and subsequently every hour until 21.30 for the determination of plasma levels of insulin, glucose, free fatty acids (FFA), and triglycerides (TG). At T = 0 (baseline 08.00 h), and 60 minutes postprandially after consumption of the three experimental meals satiety related hormones glucagon-like peptide-1 (GLP-1) active, and ghrelin-active were sampled. Adiponectin levels were sampled at T = 0, before lunch (11.30 h) and at the last blood sampling that day (21.30 h). The next morning at 08.00 h (T = 24), before subjects left the respiration chamber, fasting blood samples were taken to assess all the markers mentioned above.

Blood was collected in standard 10 ml ice-cooled vacutainer blood collection tubes containing EDTA to prevent clotting. Plasma was obtained by low-speed centrifugation within one hour after blood sampling, frozen in liquid nitrogen, and stored at -80°C until further analysis. Phenylmethylsofonyl fluoride was added to the active ghrelin plasma samples. For GLP-1 active measurements blood was mixed with 60 µl dipeptidyl peptidase ΙV inhibitor (DPP-IV) (Linco Research Inc., St Charles, Missouri, USA). Plasma insulin, active ghrelin, adiponectin, GLP-1 active concentrations were measured by radioimmunoassay (Millipore, Billerica, MA, U.S.A). Plasma glucose, FFA, and TG concentrations were measured with the use of an enzymatic colorimetric method on a Cobas Fara spectrophotometer (Roche Diagnostica, Basel, Switzerland).

### Visual Analogue Scales

Appetite profiles were measured using anchored 100-mm visual analogue scales (VAS) with words at each end that expressed the most extreme rating to measure hunger, fullness, satiety, thirst, and prospective food consumption [17]. Subjects completed these questionnaires just before, 30, and 60 minutes after consumption of the three experimental meals in the LFr diet, and the next morning at 08.00 h. At the similar time points, questionnaires were completed in the HFr diet.

### Indirect Calorimetry

The respiration chamber is a 14 m^3^ room and is furnished with a bed, chair, table, television, radio, telephone, computer, wash-bowl, intercom, and a deep-freeze toilet. Air locks are used for exchange of food and urine. EE was determined from the measurements of O_2_ consumption, CO_2_ production, and urine nitrogen excretion according to Brouwer [18]. The chamber is ventilated with fresh air at a rate of 70–80 l/min. The ventilation rate was measured with a dry gas meter (type 4; Schlumberger; Dordrecht, The Netherlands). The concentrations of O_2_ and CO_2_ were measured using a paramagnetic O_2_ analyser (OA184A; Servomex, Crowborough, UK) and an infrared CO_2_ analyzer (Uras 3G; Hartmann & Braun, Frankfurt am Main, Germany). Ingoing air was analysed one every 15 minutes and outgoing air once every 5 minutes. The gas sample to be measured was selected by a computer, which also stored and processed the data. Physical activity was monitored using a radar system, which is based on the Doppler principle. 24 h urine samples were collected in containers with 10 ml HCL to prevent nitrogen loss by evaporation. The 24 h urine nitrogen concentration was used to calculate total daily nitrogen excretion, which was measured with a nitrogen analyzer (CHN-O-Rapid; Heraeus, Hanau, Germany). 24 h EE was calculated from 08.00 hours (first morning) to 08.00 hours (second morning). Diet induced thermogenesis (DIT) was calculated by plotting EE against radar output; both averaged over 30-minutes periods. The radar output during stepping has been excluded, because it is not reliable measurement for the 24 h EE components calculation. The intercept of the regression line at the lowest radar output represents the EE in the inactive state (resting metabolic rate; RMR), consisting of DIT and SMR. DIT was determined by subtracting SMR from RMR. Activity-induced EE was determined by subtracting SMR and DIT from 24 h EE. PAI was calculated by dividing 24 h EE by SMR [19], [20].

### Body Composition

In the fasted state, body density was determined by underwater weighing for baseline characteristics. Lung volume was measured simultaneously using the helium dilution technique. Body weight was determined on a digital balance, accurate to 0.001 kg (IDI plus; Mettler Toledo, Tiel, the Netherlands). Under water, body weight was measured using a digital balance, accurate to 0.01 kg (EC240; Mettler Toledo, Tiel, the Netherlands). Lung volume was measured using a spirometer (Volugraph 2000; Mijnhardt, Bunnik, the Netherlands). Body fat percentage was calculated using the equation of Siri [21].

### Statistical Analyses

SPSS software (version 15 for windows; SPSS) was used for data entry and analysis. All data is reported as means ± standard error mean (SEM). Homogeneity of the data was checked with the Kolmogorov-Smirnov test, ln transformation was applied when data were not normally distributed. CGMS and EE data were calculated per 24 hour, during the day (8 am –23 pm) and during the night (23 pm –8 am). CGMS data, EE, substrate partitioning, and (net incremental) area under the curves ((net i)AUCs) calculated using the trapezoid method, were compared between the two intervention diets with a paired t-test. Mixed-model ANOVA [22] was used to compare the intervention diets at the different time points for VAS scales and the metabolic markers. Outcomes were corrected for multiple testing. Statistical significance was set at p<0.05.

## Results

### Subject Characteristics

Twelve healthy non-smoking men aged 23±1.2 y, with a mean body mass index (BMI) of 21.6±0.6 kg/m^2^ and with a mean percentage body fat of 14.1±1.4% participated in this study. Subject characteristics at baseline are shown in table 1.

### Energy Expenditure and Substrate Partitioning

Energy intake was similar by design in both intervention diets (12.0±0.3 MJ/d) (table 2). Total energy expenditure (TEE) and energy balance were not significantly different between both diets. The LFr diet showed a significantly higher RMR (in this case SMR + DIT) compared with the HFr diet (8.5±0.3 vs. 8.0±0.2 MJ/d respectively). SMR and DIT (p = 0.094) tended to be increased (NS) in the LFr diet. No effect on 24 h AEE, PAL and RQ was observed between the intervention diets. Protein oxidation significantly increased in the LFr diet during the day and total 24 h (106.9±7.1 vs. 90.6±4.3 g/d). No significant differences in 24 h, day and night CHO and fat oxidation were found.

### Metabolic Markers Measured at Fixed Time Points

The LFr diet showed significantly higher peaks and lower troughs for glucose and insulin levels compared with the HFr diet during the day (figure 1). The AUC of 24 h glucose was significantly lower in the LFr diet (7276.1±149.8 mmol/L) compared with the HFr diet (7664.6±184.5 mmol/L), although the AUC of insulin was not significantly different between the two diets. In general, there was a tendency for higher FFA levels in the LFr diet, in particular after dinner compared with the HFr diet (figure 1). TG profiles were significantly higher after lunch in the LFr diet, however TG levels were significantly higher in the evening in the HFr diet. GLP-1 active and adiponectin levels showed no significant differences between the intervention diets, but overall levels tended to be higher in LFr diet (figure 2). The LFr diet significantly decreased ghrelin-active levels one hour after breakfast, and showed the same trend throughout the day in the LFr diet.

### CGMS

Complete CGMS data of twelve subjects was obtained. Mean, maximum, minimum glucose concentration, and the (net i)AUCs were calculated per diet per 24 hour from the CGMS data, during the day and night and showed no significant differences between the two interventions (table 3). Nevertheless, the CGMS data clearly showed the different glycemic patterns of the two meal frequency diets (figure 3). Glycemic variability (conga 1,2,4 and CV) did not change between both intervention diets (table 3). The correlation between the CGMS data and glucose data was significant in the LFr diet (R^2^ = 0.333; p = 0.05), and not in the HFr diet.

### Appetite Measurements

At fixed time points throughout the day hunger, prospective food consumption and thirst ratings significantly reduced, and satiety and fullness ratings significantly increased in the LFr diet compared with the HFr diet (figure 4, graphs of prospective food consumption, thirst and fullness were not shown because of the same trend). The AUCs of all appetite measurements were significantly different between the two diets (only shown for hunger and satiety).

## Discussion

Increasing meal frequency resulted in significantly lower peaks, higher troughs and constant glucose (higher AUC) and insulin values compared with the LFr diet under isoenergetic well-controlled conditions in lean healthy males. Nevertheless, no effect of meal frequency was observed on substrate partitioning of CHO and fat. Protein oxidation, RMR (in this case SMR + DIT) and appetite control increased significantly in the LFr diet compared with the HFr diet.

Our results are in accordance with findings from Solomon et al. [1], who found that 2 meals per day led to greater fluctuations in glucose, insulin, and ghrelin responses (i.e. greater peaks and lower troughs) compared with the 12 meals per day assessed throughout an 8-h period. Nevertheless, the lower AUC of glucose in the LFr indicates glycemic improvements, we suggest that this can lead to a better body weight control on the long term.

The CGMS data showed the glycemic excursions and clearly indicated the differences between the two diets during the day. However, baseline values are somewhat lower than the glucose levels measured at the fixed time points. The accuracy of the sensor has been discussed and discrepancies occasionally were seen between interstitial tissue and blood glucose levels in detecting low glucose values. Therefore, the CGMS is a good method to assess patterns of glycemic excursions and not the absolute degree of glycemic excursions [15].

The higher rise and subsequently fall of insulin in the LFr diet was suggested to result in a higher fat oxidation, which was not observed in this study. These findings are in line with a review by Bellisle [23] and a recent review by Leidy et al, who discussed eating frequency and energy regulation in controlled feeding studies [4]. Those reviews also indicated that eating frequency appears to have no effect on energy expenditure. Another explanation might be that the insulin levels did not increase high enough to inhibit fat oxidation in the HFr diet. Maybe a certain threshold has to be reached before substantial inhibition will occur. The half-maximal suppression of lipolysis is seen at around 120 pmol/l (17 µU/ml) of insulin, and at the peak of insulin after a typical carbohydrate breakfast (400–500 pmol/l; 57–72 µU/ml), adipocytes lipolysis will be maximally suppressed [24]. In addition, Mandarino et al. demonstrated with euglycemic insulin infusions that basal rates of FFA and fat oxidation were suppressed by 70–80% at an insulin level of 20–25 µU/ml and were essentially completely suppressed at insulin concentrations >50 µU/ml [25]. Our data showed insulin levels between 30 and 40 µU/ml in the HFr diet, which suggests that the threshold for maximal suppression of lipolysis was not reached in these subjects.

Protein oxidation increased significantly in the LFr diet, which could be explained by body’s limited capacity to store protein. The larger portion size and thus absolute amount of protein intake at each meal in the LFr diet resulted consequently in a higher protein oxidation. We speculate that the lower protein oxidation in the HFr diet might be a relevant dietary strategy in elderly to increase daily protein uptake and preserve lean tissue, because aging is accompanied by a progressive decline in skeletal muscle mass, also known as sarcopenia [26]. Additionally, it is suggested that the postprandial rise in plasma essential amino acids concentration, particularly leucine, defines the subsequent postprandial rate of muscle protein synthesis [27]. Nevertheless, observed changes in protein metabolism on whole-body level do not necessarily represent changes on muscle level [28]. Therefore, more research is necessary to investigate effects of different meal frequencies in elderly and in particular on muscle protein synthesis.

The trend of a higher DIT (p = 0.094) and SMR in the LFr diet is translated into a significantly higher RMR. This is a relevant observation because a low RMR is considered a risk factor for weight gain leading to obesity [29]. The higher RMR in the LFr diet might have been stimulated by a plasma insulin induced increase in the activity of the sympathetic nervous system [30], [31]. Other studies reported that no changes in RMR were observed as a result of increased meal frequency [32], [33]. However, these studies investigated meal frequency at a range of 2 vs. 7, and our study investigated meal frequency at a larger range (3 vs. 14).

Consuming the LFr diet resulted in increased feelings of satiety (AUC), and more inhibition of the satiety hormone ghrelin-active after breakfast and decreased feelings of hunger (AUC) throughout the day. Hence, we suggest that the LFr diet resulted in a better appetite control, although subsequent food intake (ad libitum meals) has not been measured in present study. Therefore, the results should be interpreted with caution. On the contrary, studies examining nibbling (small, frequent meals) compared to gorging (large, few meals) under isoenergetic conditions over a range of meal frequencies from 2 to 12 meals/d provided conflicting evidence, but over a narrower range suggest there may be some tendency for a 6-meals/d pattern to improve appetite control relative to a 3-meals/d pattern [34]. A point to consider when interpreting the study findings includes the energy level of the study diets (varied from energy restriction to isoenergetic) and resulting meal portions. The differential responses between smaller and larger eating occasions may simply be due to the inability of the body to detect the size of a smaller eating occasion as an adequate physiological load, reducing or eliminating the eating-related responses typically observed when larger eating occasions occur [4].

We designed this study to investigate different meal frequencies under isoenergetic well-controlled conditions, eliminating differences in energy balance as a confounding factor. Furthermore, potential interactions with factors such as dietary composition, food form, nutritional quality, and portion size served were also minimal in this study. A disadvantage of this study design is that the changes in feelings of hunger and satiety could not result in adjustments in subsequent energy intake since the diet was not *ad libitum*. Accordingly, it is difficult to generalize these metabolic results to a daily life setting. It is unclear what will happen when subjects consume meals with a higher frequency, have *ad libitum* access to food and how this would affect total energy intake. In addition, in our study a snack was chosen to represent a smaller-sized portion of a typical meal taken more frequently throughout the day. In a free-living situation snacks are generally high-sugar or high-fat foods [1] and therefore total energy intake probably will increase.

The subjects of our study were young and healthy, therefore they have a good capacity to switch between substrates, which indicate a high metabolic flexibility. However, when subjects are overweight, obese or have type 2 diabetes their metabolic flexibility is reduced. For that reason, subjects with metabolic inflexibility could have more difficulties handling a high meal frequency diet and this would be interesting to investigate in the future.

In conclusion, glucose and insulin profiles showed greater fluctuations, but a lower AUC of glucose in the LFr diet compared with the HFr diet. The higher peaks and subsequently lower troughs of insulin in the LFr diet did not lead to a higher fat oxidation as hypothesized. RMR and appetite control increased in the LFr diet, which can be relevant for body weight control on the long term. However, this was studied for one day in young healthy males, which are very metabolic flexible. Therefore, populations at risk related to substrate partitioning and long-term effects have to be studied before firm conclusions can be made about the mechanistic effects of meal frequency on the metabolic profile and substrate partitioning.

## Supporting Information

Protocol S1 **Trial Protocol.**
(DOC)
