# Supplementary material for: Effects of Meal Frequency on Metabolic Profiles and Substrate Partitioning in Lean Healthy Males
Source: PLoS One. 2012 Jun 13;7(6):e38632. doi: 10.1371/journal.pone.0038632 (PMC3374835; doi:10.1371/journal.pone.0038632)
Supplement: Protocol S1 — Trial Protocol. (DOC) [file pone.0038632.s001.doc]

**Effect of feeding frequency on glucose and insulin metabolism and substrate partitioning**

**(25 June 2009)**

**Principal investigator (address correspondence to):**

Prof. Dr. Wim Saris

Division of Human Biology, Maastricht University

Universiteits Singel 50

Tel: 043-3881619 / 06-53641475

E-mail: w.saris@hb.unimaas.nl

**Project group:**

MJM Munsters, Department of Human Biology, NUTRIM, Maastricht University

Prof. Dr. WHM Saris, Department of Human Biology, NUTRIM, Maastricht University

**Table of content**

[Introduction 3](#__RefHeading___Toc231208232)

[Research objective 4](#__RefHeading___Toc231208233)

[*Primary objective: 4*](#__RefHeading___Toc231208234)

[*Secondary Obective(s): 4*](#__RefHeading___Toc231208235)

[Study design 5](#__RefHeading___Toc231208236)

[Ethics 5](#__RefHeading___Toc231208237)

[Publication of results 5](#__RefHeading___Toc231208238)

[Methods 6](#__RefHeading___Toc231208239)

[Subjects 6](#__RefHeading___Toc231208240)

[*Inclusion-criteria: 6*](#__RefHeading___Toc231208241)

[*Exclusion-criteria: 6*](#__RefHeading___Toc231208242)

[Procedures and measurements 7](#__RefHeading___Toc231208243)

[*Screening visit 7*](#__RefHeading___Toc231208244)

[*Protocol 7*](#__RefHeading___Toc231208245)

[*Food prepartion 9*](#__RefHeading___Toc231208246)

[Analyses 10](#__RefHeading___Toc231208247)

[Hunger and satiety questionnaire 10](#__RefHeading___Toc231208248)

[Energy expenditure 10](#__RefHeading___Toc231208249)

[Body composition 10](#__RefHeading___Toc231208250)

[Continuous glucose monitoring systems (24 hr glycemic control) 10](#__RefHeading___Toc231208251)

[Blood and urine analysis 11](#__RefHeading___Toc231208252)

[Plasma insulin 11](#__RefHeading___Toc231208253)

[Glucose 11](#__RefHeading___Toc231208254)

[Leptin 11](#__RefHeading___Toc231208255)

[Free fatty acids 12](#__RefHeading___Toc231208256)

[Triglycerides 12](#__RefHeading___Toc231208257)

[Adiponectin 12](#__RefHeading___Toc231208258)

[CRP 12](#__RefHeading___Toc231208259)

[GLP-1, Glucagon, CCK 12](#__RefHeading___Toc231208260)

[Ghrelin 12](#__RefHeading___Toc231208261)

[24h urine nitrogen 13](#__RefHeading___Toc231208262)

[Statistics 14](#__RefHeading___Toc231208263)

[Safety aspects and Risks 15](#__RefHeading___Toc231208264)

[Compensation 16](#__RefHeading___Toc231208265)

[References 17](#__RefHeading___Toc231208266)

# Introduction

The recent escalating obesity trend in man is due to an imbalance between energy intake and energy expenditure. Energy intake is influenced by the effect of food’s energy density, total energy content and feeding frequency and the extent to which these alter satiety. Of these factors, feeding frequency has received least attention. Epidemiological evidence in human subjects indicates increasing trends in recent years of dietary snacking and increased meal frequency [1,2] and such studies show positive relationships between snacking and increased energy intake and BMI [2], illustrating the potential importance of investigating feeding frequency.

A number of studies report a positive impact of increased meal frequency on factors such as lipaemia, thermogenesis and fasting glycaemia [3], whilst another study showed the opposite [4] and further data show that no differences exist [5]. The study of Rashibi et al (2003) found that low frequency feeding was associated with an increased level of glucose and a decreased level of insulin [6]. The study of Solomon et al (2008) observed that increased feeding frequency may disrupt the insulin-ghrelin relationship which may be relevant to diminish regulation of ghrelin seen in insulin resistance [7]. This is relevant to the increased snacking habits seen in our society. This study provides the only data in human subjects and therefore further work is prudent. Also given the inconclusive evidence in the literature regarding feeding frequency and its metabolic implications, very well controlled trials are required to resolve speculation that the current increase in snacking habits contribute to the escalating obesity and type 2 diabetes mellitus epidemic.

In addition, the effects of feeding frequency on 24 hr profiles of insulin and glucose responses and substrate partitioning are not currently known. Therefore, the present study investigated the effects of meal frequency during equi-energetic feeding regimes upon the responses on insulin and glucose under strictly controlled condition.

Several gastrointestinal peptides are involved in metabolic processes and are dysregulated in states of metabolic disorders [8]. One of these peptides, ghrelin, an orexigenic hormone has also been implicated in the control of fuel metabolism, appetite and pancreatic insulin release, factors quite central to the onset of metabolic disease [9]. Glucagon-like peptide 1 (GLP-1) is a 30-amino peptide hormone that is released from the intestinal L-cells into the circulation after a mixed meal [10]. The potential role of Cholecystokinin (CCK), a gastrointestinal hormone that has been shown to play an important role in satiety in human subjects [11] will also be investigated. For that reason, satiety hormones and feelings of hunger and satiety will be investigated in this experimental study with different feeding frequencies.

## Research objective

### Primary objective:

The presently proposed study aims to investigate how different feeding frequencies lead to differences in glucose and insulin metabolism on a 24 hr basis.

We hypothesize that low feeding frequency will improve glycemic and insulinemic control on a 24 hr basis compared to high feeding frequency. The 24 hr profiles of glucose and insulin will be compared between the 2 diets (AUC 24 hr).

### Secondary Obective(s):

**A:** To investigate the differences in substrate partitioning: fat and carbohydrate oxidation on a 24 hr basis after two different feeding frequencies. Outcome parameters: fat and carbohydrate oxidation (gram/24 hr) and 24 hr balance (Kj/24 hr).

**B:** To investigate the effects of feeding frequency on metabolic parameters on a 24 hr basis. Outcome parameters: plasma levels of glugacon, FFA, TG, leptin, adiponectin, CRP and satiety hormones (CCK, GLP-1 and Ghrelin). The profiles of these parameters will be compared between the 2 diets over 24 hours (AUC 24 hr).

**C:** To investigate the effect of feeding frequency on feelings of hunger and satiety on 24 hr basis. Outcome parameter: hunger and satiety scores. The hunger and satiety profiles will be compared between the 2 diets over 24 hours (AUC 24 hr).

It is expected that a low feeding frequency will increase fat oxidation compared to a high feeding frequency.

It is expected that a high feeding frequency will increase plasma levels of ghrelin, FFA, TG, CRP, glucagon and feelings of hunger compared to a low feeding frequency

Satiety, satiety hormones (CCK, GLP-1) and plasma levels of leptin, adiponectin will be lower after a high feeding frequency compared to a low feeding frequency.

# Study design

## Ethics

All subjects will be informed orally as well as in writing about the aim of the study, study objectives, protocol, privacy and insurance issues, test burden and reimbursement of expenses. There is an independent expert available for the test subjects to consult if they have any queries about the study that they do not want to discuss with the researchers themselves. All data to be collected in this study will be encoded. It will not be possible to deduct any information to individuals. The investigator ensures that this study is conducted in full conformance with the principles of the ‘Declaration of Helsinki’ (59th WMA General Assembly, Seoul, October 2008) as well as the ‘Wet Medisch Wetenschappelijk Onderzoek met Mensen’ will be applied. The investigator assures that subject’s anonymity will be maintained. On documents subjects will not be identified by their names, but by an identification code. The investigator will keep a subject identification log showing codes, names and addresses of participants, e.g. subject written informed consent forms. The investigator will maintain these documents in strict confidence. Subject materials will be coded by the identification code and will be destroyed afterwards. All biological samples, which will be obtained in this study, will be treated strictly confidential. All samples will be coded with reference numbers, which can be linked to individual data only by the main investigators (MJMM and WHMS) who have access to the code. All biological samples will be used for analytical purpose only. After finishing the analyses, the samples will be stored 5 years maximal before additional measurement related to the topic of this study. Subjects do not have to give permission again to use their samples for the additional measurements. All subjects will be informed about general results of the study, based on the calculation averages of the outcome parameters. Also each subject receives his or her individual study results. A WMO subject insurance and liability insurance are available for all subjects. Subjects can end their participation whenever they want without giving an explanation.

## Publication of results

The results of this study will be published in peer-reviewed international scientific journals. The sponsor of the study cannot delay publication of the results.

# Methods

In order to estimate how many subjects need to be recruited, power analyses are performed. To do so, results from the study of Rashidi et al [6] are used. This study examined the effects of different feeding frequency on blood lipids, glucose and insulin during 14 hours. The plasma insulin response increased from 4.1 ± 3.3 (µIU/ml) with a low feeding frequency to 7.7 ± 5.8 (µIU/ml) with a high feeding frequency (n=15).

N1 = 2 * (Zsig + Zb)2 * s2 / (µ1 - µ2) + (Zsig2 / 4)

N1: number of subjects in diet trial
Zsig: Z-score belonging to a significance of 0,05: 1.96
Zb: Z-score belonging to the power (0,95): 1.65
s: Standard deviation, based on previously found results: 5.8 (µIU/ml)
µ1 - µ2: the expected difference in insulin level during 14 hours in our study between low and high feeding frequency: 3.6 (µIU/ml)

N1 = N2 = 12

Taken into account that 10% of included subjects will drop out, we will include 14 subjects.

## Subjects

Subjects will be recruited from existing subjects-files and advertisements in both local newspapers and university billboards. Since energy expenditure declines with increasing age, a maximal age of 35 years is set to form a homogeneous adult group. Also for homogeneity reasons as well as to avoid menstrual cycles effects on energy expenditure, only Caucasian males are included. The following inclusion- and exclusion-criteria are specified:

### Inclusion-criteria:

- BMI: 18.5-25 kg/m^2^ (lean)
- Gender: Male
- Age: subjects has to be older than 18 and including 35 years old
- Caucasian

### Exclusion-criteria:

Metabolic abnormalities such as:

1. Lactose intolerant
2. Diabetes Mellitus
3. Hypertension (systolic/diastolic blood pressure >140/90)
4. Hypotension (systolic/diastolic blood pressure <90/60)
5. Cardiovascular diseases
6. Asthma and other obstructive pulmonary diseases
7. Elevated fasting blood glucose level (> 5,6 mmol/L)

## Procedures and measurements

### Screening visit

After giving informed consent, a screening visit will be planned. In the early morning, after an overnight fast, subjects will come to the University. In this visit, following standard measurements will be done:

- Body weight
- Length
- Body composition
- Blood pressure (diastolic and systolic)
- Fasting blood sampling for glucose and insulin
- OGTT

Further, subjects will complete a health questionnaire, including questions about cardiovascular diseases, asthma, medicine use and other medical issues. Also the respiration chamber will be shown to the subjects during the screening visit so they will know what to expect from the stay in the respiration chamber.

### Protocol

This study is a randomized, controlled crossover study with 1 group of 14 healthy male volunteers. Subjects are exposed to two different diets, which all consists of 30 Energy% (En%) of fat and with 55 En% of Carbohydrates and 15% of dairy protein and low GI (GI < 40). Both diets are exactly the same, they contain the same products and energy content, however only the frequency of the meals differ. The diet will be divided into 3 or 14 meals on a day. The meal contains of Cracottes natural, semi-skimmed milk, melon, tomato and olive oil.

Each subject will be given 14.3 ml/kg water to consume *ad libitum* throughout the trial. This volume of water corresponded to 1 litre per 70 kg body mass, which was considered appropriate for the intervention.

For preparation of the diets, see section food preparation.

To have a same baseline condition before each diet, subjects have to standardize the diet and activity over the 3 days before the tests. Therefore food-intake and activity diaries have to be filled out before the first test day and repeated exactly similar before other test days (see attachments for food-intake and activity diaries).

Each respiration chamber visit starts after the continuous glucose monitoring system (CGMS) placement plus preparation time at 20.00h and ends 36 hours later at 08.00h. The first twelve hours are to accustom to the respiration chamber. Then, energy expenditure measurements are made during 24 hours at a temperature of 22°C at daytime, at night temperature is set at 20°C. During the 24hr day period three block of 15 minutes of activity (stepping) will be included. Activity will be monitored with a radar system based on the Doppler principle.

Urine is collected during the respiration chamber stay from 8.00h in the morning until 8.00h next day (24 h); it is collected and stored in a portion of 24h. Both respiration chambers present at Maastricht University are used at the same time to enable social contact between the subjects. The interval between two visits is at least 1 week.

At arrival, the Continuous Glucose Monitoring System (CGMS) will be inserted in the peri-umbilical region of the subject and subjects enter the respiration chamber at 20.00 h. Next day, in the morning an indwelling cannula will be inserted into an anticubital vein for the withdrawal of the first fasting blood samples. Then subjects receive one of the two different diets randomly. The first diet exists of a breakfast at 08.00 h, a lunch at 12.00 h and dinner at 17.00 h. The second diet exists of 14 meals on one day, every hour a meal will be provided to the subjects starting from 08.00 h until 21.00 h. Energy expenditure will be measured during the day and subjects are fed in energy balance.

Blood (10 mL) will be sampled at baseline and 30 minutes after consumption of the first meal and then every hour blood will be sampled for insulin, glucose, glugacon, FFA, TG, leptin, adiponectin and CRP. At T0 (baseline 08.00) and T1, T5, T10 and T24 the satiety related hormones GLP-1, CCK and Ghrelin will be sampled (10mL). At the last day, fasting blood samples will be taken in the morning at 08.00 h before subjects leave the respiration chamber. At each test 210 ml blood will be sampled, for the complete study in total 210 ml x 2 tests + OGTT (16 ml) = 456 ml blood will be sampled.

The feeling of hunger and satiety will be assessed by means of a short standard questionnaire (see attachment D. honger en verzadigingsvragenlijst). Hunger and satiety questionnaire has to be filled out just before and 30 minutes and 60 minutes after the meals of 08.00 h, 12.00 h and 17.00 h.

Blood samples from one subject will be analyzed for insulin, glucose, glucagon, adiponectin, leptin, FFA, TG, GLP-1, Ghrelin, CCK, CRP within one run at the end of the study under strict quality control. Plasma will be obtained by low-speed centrifugation within one hour after venapuncture and stored as appropriate.

***Flow chart (see appendix)***

### Food prepartion

All food used in this study will be prepared and stored in a dietary intervention kitchen, located at Universiteitssingel 50, 2^nd^ floor, room 2.359A. This kitchen is dedicated solely to food preparation and storage for human intervention trials. The diets consist of fresh and packed products that will be bought in the convenience store. The meals will be prepared just before ingestion by MJMM and the products will be weighted on a microscale balance that is only used to weight products for human consumption. Therefore the health risks of the diets for the subjects will be reduced to a minimum.

# Analyses

## Hunger and satiety questionnaire

Hunger and satiety will be assessed by a questionnaire (see attachment D. Honger en verzadigingsvragenlijst) with questions regarding the feeling of hunger and satiety. Subjects will be asked to score feelings of hunger and satiety on a visual analogue scale (a line of 100-mm).

For the physiological part of this study, several measurements will be made: energy expenditure, fat oxidation, body composition, and 24 hr glycemic control. The methods are described in detail below.

## Energy expenditure

Energy expenditure measurements will be made in a 14 m^3^ respiration chamber (described in detail by Schoffelen et al[12]). TDEE will be determined from the subjects O_2_ consumption, CO_2_ production and N_2_ excretion. The SMR will be calculated as the lowest energy expenditure (measured in 30 minutes intervals) over three consecutive sleeping hours [12].

In the first 12 hours, the subject can accustom to the chamber and the SMR can be assessed. Based on the SMR (multiplied with a physical activity index - the ratio TDEE/SMR - of 1.55), energy balance will be determined. Subjects are fed in energy balance in all situations [13]. For standardization of the measurements, the humidity in the respiration chamber will be fixed, clothing is standardized and physical activity is prescribed by means of a standardized physical activity protocol [14]. Feelings of hunger and satiety will be recorded.

## Body composition

To be able to normalize against the metabolic active body mass, body composition is assessed. Body density is determined using underwater weighing with helium dilution [15]. Body mass is assessed in fasting conditions as a measure for energy balance in the morning.

## Continuous glucose monitoring systems (24 hr glycemic control)

Day 1

In subjects’ interstitial glucose levels in the abdominal subcutaneous tissue will be monitored continuously over 36 hours by means of a CGMS System after insertion of this system (Medtronic MiniMed). This system will be placed by means of a needle, the needle will be removed after insertion of this system. Medtronic MiniMed’s Sensor works by using the enzyme glucose oxidase to convert glucose at the Sensor surface into electronic signals. The Sensor sends these signals in a steady stream through a Cable to the Monitor. The Monitor samples the signals once every 10 seconds and records an average signal every five minutes, providing as many as 288 Sensor readings in a 24-hour period. These electronic signals, and any fingerstick blood glucose measurements entered into the Monitor, are stored in the Monitor.

Day 2.

During this day the person with the CGMS will keep close attention to the protocol and has to write down all physical activities at each specific time during the day to gain insight into the subjects’ activities and glycemic excursions during this day.

Day 3

In the morning (after 36 hours) the CGMS will be removed and the acquired data will be transferred onto a personal computer. This PC will be equipped with a special software program for the analyses of all the acquired data.

## Blood and urine analysis

Blood is collected by a venous catheter in standard 10 ml ice-cooled evacuated blood collection tubes containing ethylenediaminetetraacetic acid (EDTA). It is used to determine plasma levels of insulin, glucose, leptin, glucagon, grhelin, free fatty acids, triglycerides, adiponectin, GLP-1, CRP, CCK. The collected urine is used for Nitrogen assays.

## Plasma insulin

Plasma insulin concentrations will be measured in all samples by using an antibody-coated-tube radioimmunoassay kit (NucliLab,Ede,The Netherlands).

## Glucose

Plasma glucose concentrations will be measured in all samples with the use of an enzymatic colorimetric method on a Cobas Mira Plus spectrophotometer with a commercially available kit. (Roche Diagnostic Systems, Basel, Switzerland).

## Leptin

Leptin is known as a regulator of food intake. Subjects with larger changes in energy expenditure between the different situations might have other plasma leptin levels. Leptin levels are measured according to Simons et al [16], using a double body “sandwich” ELISA with monoclonal antibody specific for human leptin.

## Free fatty acids

Free fatty acids level is measured using the Wako NEFA C test kit.

## Triglycerides

Triglyceride concentrations were measured with an automatic analyzer using manufacturer’s reagents (PoluChem Analyser).

## Adiponectin

Adiponectin secretion is an energy balance related hormone. The study of Shand BI [17] shows that adiponectin is an important molecular link between obesity, insulin resistance and atherogenic lipoproteins. Therefore the plasma adiponectin level is measured using a RIA.

## CRP

Dietary GI was significantly and positively associated with plasma highly sensitivity (hs)-CRP concentrations [18]. hs-CRP will be analyzed with latex-enhanced immunonephelometry on a BN II analyzer (Dade Behring, Newark, DE).

## GLP-1, Glucagon, CCK

Several other insulinotropic factors are known to potentiate postprandial insulin secretion such as gastro intestional hormones such as GLP-1, Glucagon and CCK. For GLP-1 measurement blood will be collected in EDTA tubes with added depeptidl peptidase IV inhibitor. GLP-1, Glucagon and CCK concentrations will be measured in all samples by using an antibody-coated-tube radioimmunoassay kit (NucliLab,Ede,The Netherlands).

## Ghrelin

Ghrelin appears to be a hunger signal [8]. Plamsa ghrelin concentrations will be measured with commercially available human radioimmunoassy (RIA) kist (Linco Research Inc.).

## 24h urine nitrogen

To correct the energy expenditure measurements for protein metabolism, 24h urine excretion is measured from urine samples using a nitrogen analyser.

# Statistics

Glucose, insulin, energy expenditure (fat and carbohydrate oxidation), metabolic parameters (plasma levels of glugacon, FFA, TG, leptin, adiponectin, CRP and satiety hormones (CCK, GLP-1 and Ghrelin)) and the hunger and satiety questionnaires (feeling of hunger and satiety) will be compared between the two different diets on a 24 hr profiles (AUC 24 hr) in the group (paired t-test). Statistical significance will be set at p<0.05.

# Safety aspects and Risks

Risks as the result of participation in this experiment are minimal. Venapunctures can occasionally cause a local haematoma or bruise to occur. Some participants report some pain during venapuncture. Insertion of the CGMS could induce some pain, because this system will be placed by means of a needle. The needle will be removed after insertion of this system. however no discomfort is expected from carrying this device. Previous studies have shown that wearing the CGMS does not hinder the subject in his normal functioning. Also participants will be asked if they are claustrophobic, because this could become a problem when they are staying in the respiration chamber and will be excluded if no solution can be found.

No harm from the dietary intervention is to be expected. All diets used during this study are purchases in the local supermarket and used before indicated expiring dates. During the test days meals will be prepared in the kitchen of the department of Human Biology which is solely dedicated for preparing of food for human use.

# Compensation

Participants will receive a compensation of **150 euro** when completing all two tests. When participants stop their contribution with this study they will receive compensation in proportion.

# References

1. Samuelson G. Dietary habits and nutritional status in adolescents over Europe. An overview of current studies in the Nordic countries. Eur J Clin Nutr 2000; 54, S21-S28.
2. Berteus-Forslund H, Torgerson JS, Sjostrom L, Lindroos AK. Snacking frequency in relation to energy intake and food choices in obese men and women compared to a reference population. Int J Obes Relat Metab Disord 2005;29, 711-719.
3. Bertelsen J et al. Effect of meal frequency on blood glucose, insulin, and free fatty acids in NIDDM subjects. Diabetes Care 1993 16, 4-7.
4. Farshchi HR, Taylor MA, Macdonald IA. Regular meal frequency creates more appropriate insulin sensitivity and lipid profiles compared with irregular meal frequency in healthy lean women. Eur J Clin Nutr 2004 58,1071-1077.
5. Verboeket-van de Venne WP, Westerterp KR. Frequency of feeding, weight reduction and energy metabolism. Int J Obes Relat Metab Disord 1993 17, 31-36.
6. Rashidi MR, Mahboob S, Sattarivand R. Effects of nibbling and gorging on lipid profiles, blood glucose and insulin levels in healthy subjects. Saudi Med J. 2003 24, 945-8.
7. Solomon TPJ, Chambers ES, Jeukendrup AE, Toogood AA, Blannin AK. The effect of feeding frequency on insulin and ghrelin responses in human subjects. Brit J Nutr 2008 100, 810-819.
8. Woods SC. Gastrointestinal satiety signals I. An overview of gastrointestinal signals that influence food intake. Am J Physiol Gastrointest Liver Physiol 2004;286,G7-G13.
9. Kojima M, Hosoda H, Date Y, Nakazato M, Matsuo H, Kangawa K. Ghrelin is a growth-hormone-releasing acylated peptide from stomach. Nature 1999 402, 656-660.
10. Holst JJ. Glucagonlike peptide 1: a newly discovered gastrointestinal hormone. Gastroenterology. 1994;107:1848-55.
11. Lieverse RJ, Jansen JBMJ, Masclee AAM and Lamers CBHW. Satiety effects of cholecystokinin in humans. Gastroenterology 1994;106:1451-54.

12. Schoffelen PF, Westerterp KR, Saris WH, Ten Hoor F (1997) A dual-respiration chamber system with automated calibration. J Appl Physiol 83: 2064-2072.

13. Schrauwen P, van Marken Lichtenbelt WD, Westerterp KR (1997) Energy balance in a respiration chamber: individual adjustment of energy intake to energy expenditure. Int J Obes Relat Metab Disord 21: 769-774.

14. Westerterp-Plantenga MS, van Marken Lichtenbelt WD, Strobbe H, Schrauwen P (2002) Energy metabolism in humans at a lowered ambient temperature. Eur J Clin Nutr 56: 288-296.

15. Ellis KJ (2000) Human body composition: in vivo methods. Physiol Rev 80: 649-680.

16. Simons JPFHA, Schols AMWJ, Campfield LA, Wouters EFM, Saris WH (1997) Plasma concentration of total leptin and human lung-cancer-associated cachexia. Clinical Science 93: 273-277.

17. Shand BI, Scott RS, Elder PA, George PM. Plasma adiponectin in overweight, nondiabetic individuals with or without insulin resistance. Diabetes Obes Metab 2003;5:349-53.

18. Liu S, Manson JE, Buring JE, Stampfer MJ, Willett WC, Ridker PM. Relation between a diet with a high glycemic load and plasma concentrations of high-sensitivity C-reactive protein in middle-age women. Am J Clin Nutr 2002;75:492-8.
